# A Novel Decoupled Feature Pyramid Networks for Multi-Target Ship Detection

**DOI:** 10.3390/s23167027

**Published:** 2023-08-08

**Authors:** Wentao Xue, Maozheng He, Yincheng Zhang, Hui Ye

**Affiliations:** College of Automation, Jiangsu University of Science and Technology, Zhenjiang 212100, China; 15516921491@163.com (M.H.); 211210301224@stu.just.edu.cn (Y.Z.); yehuicc@just.edu.cn (H.Y.)

**Keywords:** multi-target ship recognition, feature decoupling module, gating attention module, global features

## Abstract

The efficiency and accuracy of ship detection is of great significance to ship safety, harbor management, and ocean surveillance in coastal harbors. The main limitations of current ship detection methods lie in the complexity of application scenarios, the difficulty in diverse scales object detection, and the low efficiency of network training. In order to solve these problems, a novel multi-target ship detection method based on a decoupled feature pyramid algorithm (DFPN) is proposed in this paper. First, a feature decoupling module is introduced to separate ship contour features and position features from the multi-scale fused features, to overcome the problem of similar features in multi-target ships. Second, a feature pyramid structure combined with a gating attention module is constructed to improve the feature resolution of small ships by enhancing contour features and spatial semantic information. Finally, a feature pyramid-based multi-feature fusion algorithm is proposed to improve the adaptability of the network to changes in ship scale according to the contextual relationship of ship features. Experiments on the multi-target ship detection dataset showed that the proposed method increased by 6.3% mAP and 20 FPS higher than YOLOv4, 7.6% mAP and 36 FPS higher than Faster-R-CNN, 5% mAP and 36 FPS higher than Mask-R-CNN, and 4.1% mAP and 35 FPS higher than DetectoRS. The results demonstrate that the DFPN can detect multi-target ships in different scenes with high accuracy and a fast detection speed.

## 1. Introduction

**Figure 1:**
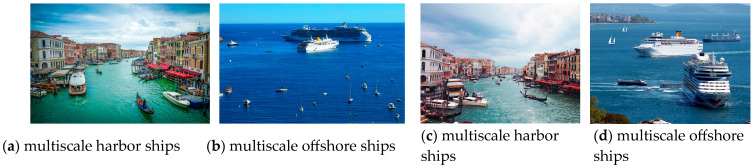
Examples of various types of ship targets.

With the development of the shipping industry, the traffic in the harbor and its surrounding waters is becoming more and more congested. The rapid growth in harbor traffic has led to marine traffic accidents occurring frequently. Ships detection and classification is of great practical value to marine surveillance in the harbor environment [1,2]. According to the imaging types for ships, there are mainly synthetic aperture radar images (SAR), spaceborne optical images (SOI), and visible images. Although SAR has the advantages of a wide field of vision and all-day and all-weather detection, and SOI has a high spatial and temporal resolution, the acquisition and preprocessing of radar images and remote sensing images require a lot of time and resources [3,4]. Compared with other categories, visible images are generally more easily obtained and have more feature information and higher resolution [5]. This paper focuses on the classification detection and position of ships in visual images, which is more practical in harbors or offshore management. However, due to the optical imaging factors, cloud cover, water fluctuation, diverse small ships, and other interferences shown in Figure 1, it becomes difficult to extract various types of ship targets from complex backgrounds, which increases the processing time and even causes missed and false detections. Therefore, it is very meaningful to identify multi-target ships in harbors or offshore quickly and accurately.

Traditional target detection mainly uses Histogram of Gradients (HOG) [6], Haar-like [7], and Support Vector Machine (SVM) methods [8] to handle ship detection tasks by extracting the shape features and color features of ships. Among the existing detection algorithms, Shi et al. [9] proposed a ship detection method based on HOG feature extraction by amplifying the separability between the ship and the background. Yu et al. [10] combined the AdaBoost classifier with Haar-like features to improve the contrast between the ship and the ocean image. Li et al. [11] used the ship head features in the transformed domain of polar coordinates for the classification of support vector machines to improve the accuracy of ship detection. However, the performance of these algorithms depends mainly on the designed features description of the ship targets, while the overall operation efficiency is governed by the qualities of feature extraction and classifiers [12,13,14]. In addition, although the traditional machine learning algorithms are relatively strong in classification, they are weak in generalization, and lack adaptability to environmental changes and multi-scale features of the targets [15,16].

In comparison to traditional machine learning methods, convolutional neural networks (CNNs) offer faster operation and greater algorithm generalization abilities. Many fruitful results have been achieved by using various methods for ship detection, such as the YOLO series, Faster-R-CNN series, DAPN, DS-CNN, HR-SDNet, and adversarial generative networks (GAN), among others [17,18,19,20,21]. Gao et al. [22] proposed the threshold attention module (TAM) based on YOLOv4 to mitigate adverse effects from complex backgrounds and noise. The detection results demonstrated an enhanced ability for multi-scale target detection. Nie et al. [23] proposed a Mask R-CNN-based ship detection method based on an attention mechanism by adjusting the weights of each channel and feature pixel. The method can significantly improve the accuracy of ship detection and segmentation. Cui et al. [24] introduced a dense attention pyramid network (DAPN) and detected multi-scale ships in different scenes of SAR images. Rabbi et al. [25] optimized low-resolution ship features by augmenting edge super-resolution with generative adversarial networks (GAN) and improved ship detection accuracy. Zhao et al. [26] developed a novel attention receptive pyramid network to highlight ship texture features and improved ship detection accuracy in the complex background.

Although most CNN-based ship detection algorithms mentioned above are effective for ship detection in general environments, they often suffer either from lack of robustness in detecting small targets, or from very expensive computational costs in detecting multi-scale ships [27]. Firstly, the surroundings of harbor ships are usually more complex than those of offshore ships because of the complex surroundings. Since small ships occupy fewer pixels in an image, the loss of detailed information easily leads to a decline in detection performance. Some of these algorithms often fail to adequately consider the scale of small targets, resulting in lower detection accuracy for small-sized ships. Additionally, waves and buildings might appear similar to ships. The presence of multiple interfering objects and complex background interference in the marine environment poses a significant challenge to the accurate detection of multi-target ships. It might cause much disturbance for these algorithms to locate and discriminate multi-scale ships accurately. Moreover, the dense distribution of multi-scale ships, the similarity of ship characteristics, and interference of background features all have a large impact on ship detection. Meanwhile, most of the research in previous works have focused primarily on the average accuracy of multi-scale ship detection, while less attention has been paid to small ship detection. In order to solve these problems, a novel decoupled feature pyramid network algorithm (DFPN) is proposed for the detection of multitarget ships in complex scenes. The main contributions of this paper are as follows.

Firstly, a feature decoupling module is developed to refine the mixed ship features into contour features and position features for solving the feature similarity problem of multi-target ships. Also, the classification and localization weights of the feature decoupling module are changed to lessen the influence of background information on Non-Maximum Suppression (NMS) scores, hence enhancing ship recognition accuracy.

Secondly, in order to overcome the problem of accuracy degradation caused by the change in target size, a feature pyramid structure based on attention (FPA) mechanism is proposed to deal with the diversity of ship targets. The channel and spatial attention modules were applied in the network to efficiently learn semantic features and contour features, and highlight small target ship features. Meanwhile, the gate of attention module can prevent the overfitting caused by the complexity of the convolution network. The proposed feature pyramid structure can reduce the effect of target size variations and improve the recall of target detection by fusing the important features of multi-scale ships.

Finally, a multi-feature fusion objective function is designed to solve the problem of difficult matching between textural features and semantic features. The method changes the method of the feature pyramid to simply splicing multi-dimensional features. It can improve multi-objective ship detection by strengthening the connection between textural features and semantic features according to the internal relationship of the ship features.

The subsequent sections of this paper are structured as follows. Section 2 presents the detail of our proposed method. The detection performance of our proposed network is evaluated and compared with other state-of-the-art methods in Section 3. Finally, the conclusions are drawn in Section 4.

## 2. Methodology

### 2.1. Network Architecture

**Figure 2:**
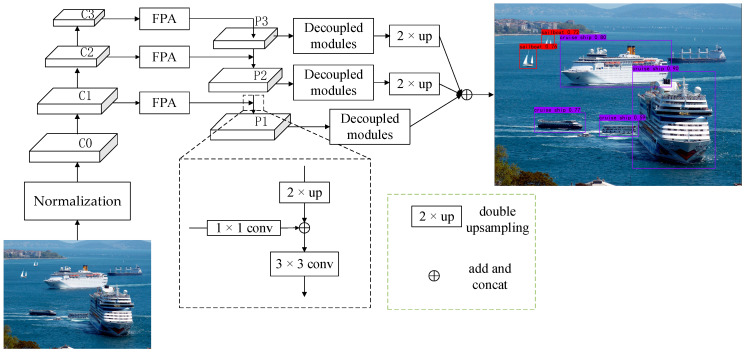
DFPN network framework diagram.

In order to adapt to multi-target ship detection in complex environments and to balance the accuracy and speed of the detection algorithm, we propose a decoupled feature pyramid network (DFPN). The overall structure of the DFPN is shown in Figure 2. It consists of a feature extraction network (ResNet-50) [28,29], a feature pyramid module combined with attention mechanism (FPA), and a feature decoupling module. Considering various geometrical shapes of multi-scale ships and complex surroundings in images, it is essential to enhance the relationships of local features with their global dependences and highlight significant information [30,31].

ResNet-50 is a deep residual network composed of 50 layers and commonly employed as a feature extraction network. It tackles the challenge of vanishing gradients in deep neural networks through the incorporation of skip connections or residual connections. The formulation for a single residual block can be expressed as:

(1) $$y=F\left(x,\left\{W_{i}\right\}\right)+x$$

where x represents the input feature map of a residual block, y denotes the output of the block, and F is the residual function that models the transformation applied to the input feature map. It is composed of a sequence of convolutional layers with weights $W_{i}$. The skip connection allows the direct addition of the input feature map x to the transformed feature map $F\left(x,\left\{W_{i}\right\}\right)$.

FPA integrates channel and spatial attention modules to extract salient feature maps with the goal of boosting regions of ship targets and suppressing the interference of complex backgrounds. In order to refine the extracted features, the feature decoupling module is designed to alleviate the inconsistency between classification and regression. Thus, the detection performance can be effectively improved. In DFPN, *C*{*x*} (*x* = 0, 1, 2, 3) is the multi-layer feature map extracted by ResNet-50. *P*{*x*} (*x*= 1, 2, 3) is the different level of fine-grained feature maps of the lateral connection. The segmentation map *P*{*x* + 1} is inferred from the enhanced feature via a 1 × 1 convolutional layer and a two-times up-sampling operation. After an element-wise addition and a convolution with a 3 × 3 kernel, the fine-grained feature map *P*{*x*} is acquired. *P*{*x*} is fed into the decoupled classification and regression network to obtain multiple NMS scores, and the best network model is obtained by discarding redundant information. In the next section, every module of our method is described in detail.

### 2.2. Approach

#### 2.2.1. Feature Decoupling Module Based on Additive NMS Scores

Feature decoupling modules achieve feature decoupling by decomposing the input feature map. Assuming the input feature map is x, the decoupling operation can be represented as:

(2) $$x=\left[x_{1},x_{2},\ldots ,x_{n}\right]$$

where *x* represents the decomposed sub-feature maps. Each sub-feature map is processed independently and the corresponding features can be expressed as:

(3) $$y_{i}=F_{i}\left(x_{i},W_{i}\right)$$

Finally, the processed sub-feature maps $y_{i}$ are merged to obtain the final output feature map y. This decoupling operation helps the network better capture multi-scale and multi-level feature information.

Many existing ship identification networks use Intersection over Union (IoU) as detection criterion. However, IoU performs well for binary classification tasks, but struggles with multi-target ship detection. To tackle the ship contour and position feature interference problem in network training, a modified scoring criterion is utilized to reduce redundant detection boxes and acquire accurate contour and position features. Thus, ship identification is divided into classification and regression tasks. The probability of the network predicting a specific ship is used as the identification criterion for the classification score (cls), and the merge of the predicted bounding box and the ground truth bounding box is used as the regression task criterion for the localization score. Finally, the scoring metric is calculated by multiplying the classification and localization scores. The conventional score criterion is represented as:

(4) $$F_{1}=I\times C$$

where $C=\left[C_{1},C_{2},\cdot \cdot \cdot ,C_{n}\right]$, $C_{i}\in \left[0,1\right]$ represent the total number of within-category categories. The scalar $I\in \left[0,1\right]$ denotes the IoU value.

To solve the problem of NMS score ranking disorder caused by high location scores in background samples, the proportion of classification scores is increased and location scores are reduced in the NMS score. The formula for the additive NMS score is as follows:

(5) $$F=\left(1-\lambda \right)I+\lambda C$$

where $\lambda \in \left[0,1\right]$ represents the weight of the category score in the NMS score value. 

**Figure 3:**
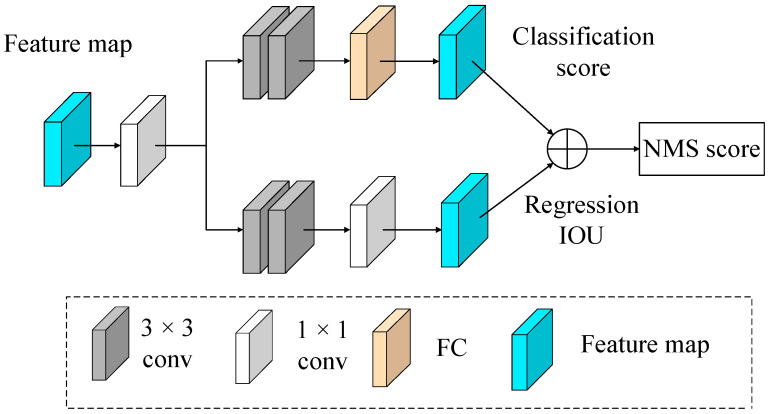
Feature decoupling diagram based on additive NMS scores.

As shown in Figure 3, the white 1 × 1 convolutional layer extracts meaningful image features, which increases network channels and refines local features. The brown 3 × 3 convolutional layer is expected to adjust the number of channels to compress the feature maps and reduce the amount of calculation. The yellow fully connected (FC) layer is used for the classification task of ship recognition. Similarly, after a 1 × 1 convolutional layer and two 3 × 3 convolutional layers, the white 1 × 1 convolutional layer is used for logistic regression to locate the predicted bounding boxes. In the classification task, the network predicts the probability of a ship as a classification score. In the regression task, the Intersection over Union between the predicted and ground-truth boxes is used as a localization score. Finally, the classification score and localization score are combined to obtain the NMS score. By training the refined contour features and positional features separately in the network, the resolution of different types of ships is improved, enhancing the network’s ability to recognize multiple ships with similar features.

**Figure 4:**
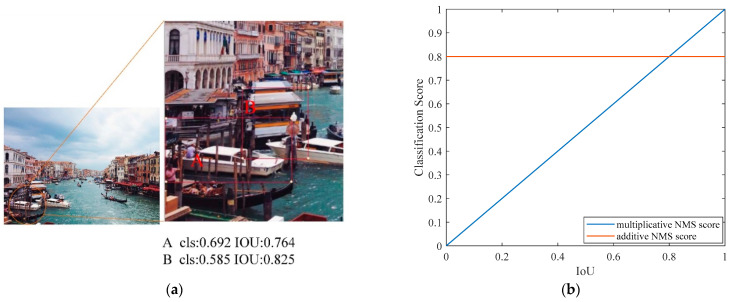
Addition NMS score analysis chart. (**a**) cls and IoU score. (**b**) NMS score.

As shown in Figure 4a, the red box A represents the target vessel, while the red box B represents interference. The IoU of the red box B is 0.061 higher than that of the red box A. This is because the labels in the dataset only annotate the ship targets and do not provide negative samples with background position features. Therefore, the training of the logistic regression localization task only includes positive samples. During the training process, negative samples may appear in the classification task, which may lead to a high localization score of negative samples in the classification network. When the localization score of the background exceeds the ship localization score, some negative samples may have a higher ranking than positive samples, thereby affecting the detection performance.

As illustrated in Figure 4b, the blue curve reflects the weight variation of classification scores in multiplication-based NMS scores, while the orange curve represents the weight variation of classification scores in addition-based NMS scores. The weight of the IoU is positively connected with the classification score in the multiplication-based NMS score curve. This means that higher localization scores have a larger influence on categorization scores. False detections induced by high localization scores in negative samples can be decreased by increasing the proportion of classification scores in the NMS score. However, the addition-based NMS score can reduce the influence of the IoU on the NMS scores. As the IoU changes, the NMS scores remain unchanged. This indicates that the proportion of localization scores in the addition-based NMS scores diminishes. Therefore, the feature decoupling module introduces an additive-based NMS score to reduce the effect of IoU on the NMS score, thus overcoming the problem of false detection due to the high localization score of negative samples.

#### 2.2.2. Feature Pyramid Module with Gated Attention Mechanism 

Because small ships have poor feature representation in multi-target ship detection, it is critical to improve their feature data. To highlight the multi-scale features of ships and improve the recognition of small ships, a gated convolutional attention module is introduced into the feature maps derived by the backbone network.

**Figure 5:**
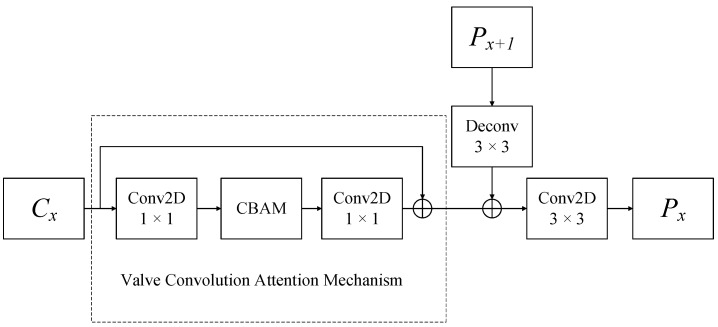
Feature pyramid model with gated attention mechanism diagram.

Figure 5 shows the structure of the FPA in detail. *Cx* refers to a basic feature map. P_x+1_ is the upper layer feature map of Px. The fine-grained feature map Px is acquired after the valve convolution attention module and deconvolution fused P_x+1_ followed by a 3 × 3 2-D convolution. *Cx* is a reduced dimension by a 1 × 1 convolutional kernel. Next, the ship contour characteristics and semantic features are highlighted using CBAM. In order to facilitate the fusion of multidimensional features, the number of channels in the input features is modified using a 1 × 1 convolutional to match the number of channels in the deconvolved P_x+1_. Finally, a 3 × 3 two-dimensional convolutional kernel is used to reduce the network parameters and obtain a fine-grained feature map Px. To prevent overfitting caused by network complexity, a gate is designed before the CBAM module. If the attention module fails to reduce the loss function, the weight parameters of the CBAM module will be updated to 0 through backpropagation. Therefore, the CBAM module will disconnect and fuse features for deconvolution. The formula for the FPA module is as follows:

(6) $$P_{3}=CBAM\left(C_{3}\right)+C_{3}$$

(7) $$C_{x\_mid}=CBAM\left(C_{x}\right)+C_{x}$$

(8) $$P_{x}=Conv\left(concat\left(C_{x\_mid},Deconv\left(P_{x+1}\right)\right)\right)$$

where *C_x_* (x = 0, 1, 2, 3) is the multi-layer feature map extracted by ResNet-50, *P_x_* (x = 1, 2, 3) is the fine-grained feature maps of the different level lateral connection, $C_{x\_mid}$ is the feature map of $C_{x}$ processed by the convolutional attention residual module, concat denotes the feature-fusion function, Deconv denotes the deconvolution operation, and Conv denotes the convolution operation.

The valve convolution attention module contains channel attention, spatial attention, and an attention module control switch. The channel attention focuses on selecting the appropriate scale of feature maps, while spatial attention focuses on the small target ship area. The combination of these two attention mechanisms can alleviate missed and false detections of small ships. Moreover, the gate of the CBAM [32] module can effectively avoid the overfitting caused by network complexity. When the attention module causes the network to overfit, the loss function of the network training grows and loss function backpropagation occurs, resulting in the automatic disconnection of the attention module. Although shallow networks can extract contour features, they lack semantic features. The deep network can extract rich semantic information, but it lacks the features of accurate location information. Therefore, FPA utilizes channel attention to concentrate on the semantic features of the ship, while spatial attention focuses on the contour features of the ship. The attention-enhanced contour features are then fused with deep semantic features. The formula of the CBAM gate is as follows:

(9) $$\begin{array}{l} M_{c}=sigmoid\left(H\left(Maxpool\left(F\right)\right)+H\left(Avgpool\left(F\right)\right)\right)\\ F_{1}=F⊗M_{c}{}^{T} \end{array}$$

(10) $$\begin{array}{l} M_{s}=sigmoid\left(H\left(Maxpool\left(F_{1}\right)\right)+H\left(Avgpool\left(F_{1}\right)\right)\right)\\ F_{2}=F+F_{1}⊗M_{s}{}^{T} \end{array}$$

where *H* is the shared multi-layer perceptron, $M_{c}{}^{T}$ and $M_{s}{}^{T}$ are transpositions of the attention mechanism weights *M_c_* and *M_s_*, and ⊗ is the inner product symbol.

#### 2.2.3. Multi-Feature Fusion Algorithm 

Numerous extracted features are simply concatenated to create high-dimensional features in the majority of fine-grained classification techniques, ignoring the natural relationships between the features. A feature regularization method is proposed in this paper to generate fine-grained ship features utilizing feature pyramid fusion and contextual information. Here, $X=\left[x_{1}^{1},\cdots ,x_{i}^{j},\cdots ,x_{N}^{M}\right]$ denotes *M* features obtained from *N* images. This fusion layer can be expressed as:

(11) $$Y_{F}=\sigma \left(\sum_{m=1}^{M}W_{P}^{m}Y_{P}^{m}+B_{p}\right)$$

where $Y_{F}$ and $Y_{P}^{m}$ denote the fusion layer and feature extraction layer of the deep neural network architecture, $W_{P}^{m}$ represents the trainable parameters of the final feature extraction layer, and sigma corresponds to the rectified linear unit activation function. To effectively incorporate multiple features, a regularized objective function is designed to achieve the optimal feature fusion. It can maintain regularization throughout the process. The objective function is defined as follows:

(12) $$\begin{array}{cc} \underset{W,\psi }{\min}=L+\frac{\lambda_{1}}{2}\left(\sum_{l=1}^{P}\sum_{m=1}^{M}{\left\|W_{l}^{m}\right\|}_{F}\right)+\frac{\lambda_{2}}{2}tr\left(W_{P}\psi^{-1}W_{P}^{T}\right) & s.t.\psi \geq 0 \end{array}$$

where L is the loss function, the coefficient matrix $W_{P}$ captures the weights of all features, and the matrix ψ characterizes the correlation between them. Notably, a higher value of ψ indicates a stronger association between the features, while a smaller value indicates a weaker association between different features. The contributions of the different regularization terms are controlled by the coefficients $\lambda_{1}$ and $\lambda_{2}$, respectively.

The optimization pipeline is summarized in Algorithm 1.
**Algorithm 1** Training of multi-feature fusion algorithm$1:\;\mathrm{Input}:\;x_{n}^{m}$$\;\mathrm{represents}\;\mathrm{the}\;m-\mathrm{th}\;\mathrm{feature}\;\mathrm{for}\;\mathrm{the}\;n\mathrm{th}\;\mathrm{picture},\;y_{n}$ is the label of the *n*th picture 2: **Pre-training:** $\;\;\mathrm{DFPN}\;\mathrm{fine}-\mathrm{tuning}\;\mathrm{and}\;\mathrm{randomly}\;\mathrm{initialize}\;W_{l}^{m}$, ψ=IMM$,I_{M}$ as the identity matrix 3: **for** epoch 1 to K do
$4:\;\;\mathrm{By}\;\mathrm{estimating}\;\mathrm{the}\;\mathrm{gradient}\;G_{l}^{m}$, the prediction error is backpropagated from layer *l* to layer 1, $5:\;\;\mathrm{weight}\;\mathrm{parameter}\;W_{l}^{m}$$\;\mathrm{of}\;\mathrm{each}\;\mathrm{layer}\;\mathrm{is}\;\mathrm{updated}\;\mathrm{as}:\;W_{l}^{m}=W_{l}^{m+1}-\gamma W_{l}^{m}-\eta G_{l}^{m+1}$ 6:  Update feature relationship matrix ψ: $\;\;\;\;\;\;\;\;\;\;\;\;\;\;\psi =\frac{{\left(W_{P}^{T}W_{P}\right)}^{\frac{1}{2}}}{tr\left({\left(W_{P}^{T}W_{P}\right)}^{\frac{1}{2}}\right)}$ $7:\;\;\mathrm{Output}:\;\mathrm{The}\;\mathrm{training}\;\mathrm{parameters}\;\mathrm{of}\;\mathrm{the}\;\mathrm{deep}\;\mathrm{architecture}\;W_{l}^{m}$ 8: **end for**

As shown in the table above, $\left(W_{P},\psi \right)$ form a coupled parameter pair. Thus, a variable optimization method is employed to iteratively optimize $W_{l}^{m}\left(l=1,\cdots ,L,m=1,\cdots ,M\right)$ and ψ separately to minimize the objective function. We first fix ψ and consider the minimization of $W_{l}^{m}$. The weight matrix for the l-th layer and the m-th feature is updated as:

(13) $$W_{l}^{m}=W_{l}^{m+1}-\gamma W_{l}^{m}-\eta G_{l}^{m+1}$$

where η is the step size of the gradient descent, and $G_{l}^{m}$ is the gradient with respect to $W_{l}^{m}$. When $W_{l}^{m}$ remains constant and other variables remain unchanged, the objective function is minimized based on ψ. Equation (13) is transformed as:

(14) $$\begin{array}{cc} \underset{\psi }{\min} & tr \end{array}\left(W_{P}\psi^{-1}W_{P}^{T}\right),s.t.\psi \geq 0,tr\left(\psi \right)=1$$

#### 2.2.4. Weighted Loss Function

In multi-target ship detection, the background information is considered as negative samples, and ship targets are defined as positive samples. Regions with small ships and high-density ships are marked as difficult samples, and regions with clear pixels and prominent ship features are labeled as easy samples. To tackle the imbalance in positive/negative classification and easy/difficult sample classification, a weighted loss function is designed to solve the class imbalance in the ship category. In the loss function,α is the weight factor of the positive and negative samples in the loss function, and β is the weight factor between the easy and difficult samples. This approach can effectively suppress background noise and overcome interference from clutter such as small ships. The weighted loss function can be represented as:

(15) L=−1N∑iN∑jMα1−pi,jβyi,jlogpi,j+1−αpi,jβ1−yi,jlog1−pi,jpi,j=eyi,j∑i,jeyi,j

In Formula (15), N represents the total number of samples in a single training batch, and M represents the number of ship categories. Meanwhile,$y_{i,j}=1$ means that the *j*-th class of ship target for the *i*-th sample is true, while $y_{i,j}=0$ means that the *j*-th class of ship target for the *i*-th sample is false. Additionally,$p_{i,j}$ indicates the probability that sample *i* belongs to class j of ship target. Normally, the weighting factor for positive and negative samples is set to $\alpha \in \left(0.65,0.85\right)$, while the weighting factor for easy and difficult samples is set to $\beta \in \left(2,5\right)$.

Formula (15) yields

(16) $$L_{1}=\alpha {\left(1-p_{i,j}\right)}^{\beta }y_{i,j}\log p_{i,j}+\left(1-\alpha \right){\left(p_{i,j}\right)}^{\beta }\left(1-y_{i,j}\right)\log\left(1-p_{i,j}\right)$$

**Figure 6:**
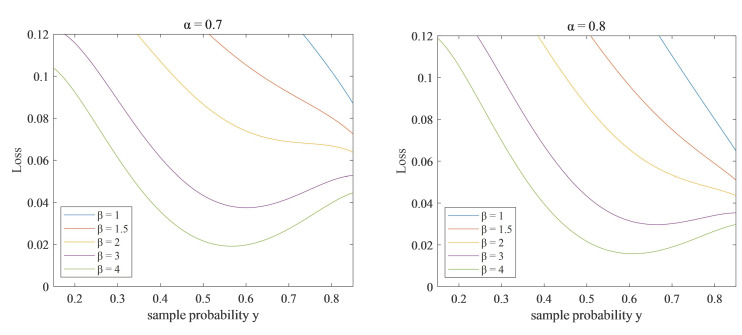
Loss function comparison graph. (**a**) Loss function at α = 0.7. (**b**) Loss function at α = 0.8.

Formula (16) demonstrates that the L1 loss function is minimized when $p_{i,j}=y_{i,j}$. Figure 6 depicts a comparison experiment of the $L_{1}$ loss function with the setting of α=0.7 and α=0.8. Notably, when β=3,β=4, the network will overfit. Based on the minimal loss function, the optimal values of α and β are 0.8 and 2, respectively. Therefore, these values are the superior choices for this experiment.

## 3. Experiments and Results

### 3.1. Dataset Description and Parameter Setting

**Figure 7:**
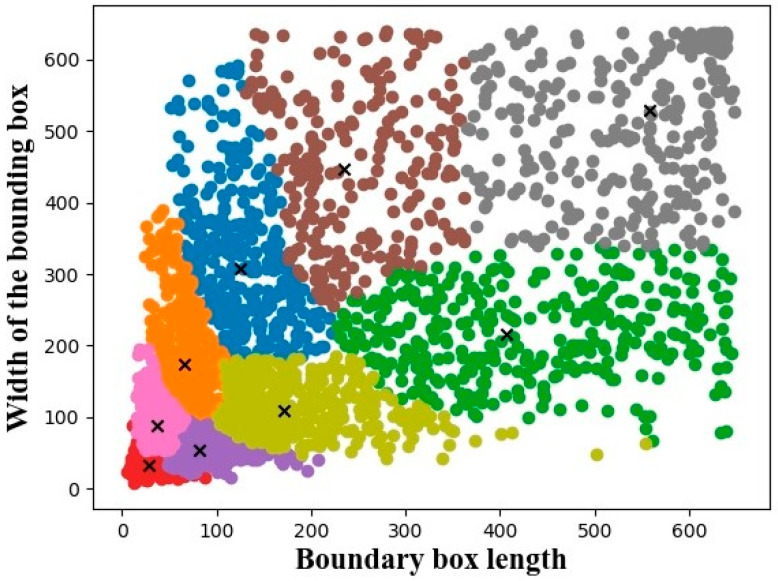
Distribution of the bounding box sizes in the ship detection dataset. The black x represents the center point of each class in the clustering algorithm.

The ship detection dataset is a visual image dataset obtained from the Kaggle website (https://www.kaggle.com/datasets/clorichel/boat-types-recognition, accessed on 10 November 2018). The dataset selected the ship images on harbor and offshore. There are four types of ships in the dataset: cruise, sailboat, gondola, and kayak. It has a size of 680 MB and contains 1213 images with 3098 multi-scale ships. The average number of ships per image is 2.55, and the resolutions of these images range from 0.5 to 20 m. The distribution of the bounding box sizes is shown in Figure 7. The heights and widths of the bounding boxes range from about 1 to 370 pixels and 1 to 380 pixels, respectively. The positions of the ships in the images are randomly distributed.

In the ablation experiment and comparison experiment, the ship detection dataset was randomly divided into a training set, validation set, and test set with a ratio of 8:1:1. Data enhancement techniques such as horizontal flipping, random cropping, and light–dark contrast conversion were used to collect 47,750 training images. These images were resized to the scale of 416 × 416 in the experiments.

All experiments were conducted on GPU (GTX 2080ti) using Pytorch 1.2.0, CUDA 10.0, and CUDNN 7.6.5. ResNet-50 pretrained was adopted as the backbone network in our method. In CBAM, the rates of both MLP in the channel attention module and convolutional layers in the spatial attention module were set to 8. To remove the interference from irrelevant bounding boxes, the K-means method was introduced to obtain anchor boxes (i.e., potential ships). Nine anchor boxes were set for small targets with dimensions of [15.9, 20.5], [24.7, 60.5], and [37.7, 29.0]; medium targets with dimensions of [45.8, 127.3], [75.7, 44.2], and [101.6, 228.3]; and large targets with dimensions of [128.2, 82.3], [252.8, 145.2], and [344.7, 339.6]. The threshold of NMS and the IoU share parameter was set to 0.5 and 0.8, respectively. In the loss function, the weighting factor of the positive and negative sample parameter was set to 0.8, and the hard and easy sample parameter was set to 2. The network was trained in two phases, each with 50 epochs and eight batches per epoch, and the optimizer was selected as the adaptive estimation optimizer (Adam). The initial learning rates of the weights were 0.001 and 0.0001 at the two stages and decayed by five times per epoch. 

### 3.2. Evaluation Criteria

In order to evaluate the performance of the proposed network and validate the effectiveness of the network model, this paper adopts precision, recall, F1-Score (F1), mAP, and FPS as the evaluation index in ship detection [33,34,35]. The calculation formula of these indicators is as follows:

(17) Precision=TPTP+FP

(18) Recall=TPTP+FN

(19) F1=2Precision×RecallPrecision+Recall

(20) $$mAP=\frac{1}{n}\int p\left(r\right)dr$$

(21) FPS=1Tper_img

In these formulas, TP, FP, and FN refer to the numbers of true positives, false positives, and false negatives, respectively. Precision rate refers to the proportion of ground truth ships predicted by the networks, and Recall rate refers to the proportion of ground truth ships predicted by networks in the total truth ships. Both of them range between 0 and 1. F1 Score is the harmonic mean of precision and recall used to evaluate networks performance. Moreover, mAP is the mean area under the precision–recall (PR) curve, where p(r) is the curve function and n is the number of ship types. Finally, FPS is the quantity of pictures detected per second, and Tper_img is the inference time cost of a method when processing an image.

### 3.3. Ablation Experiments

Ablation experiments were conducted on a ship detection dataset to verify the functionality and contributions of each component. The experimental results are presented in Table 1. The FPN consists of a feature extraction network (ResNet-50) and a feature pyramid. In addition, FPN1 is the FPN with the feature decoupling module, and FPN2 is the FPN1 with the FPA architecture. In Table 1,, FPNX (X = 1, 2, …,6) indicates the modules added on top of the FPN using the “√” symbol. The scores of all indicators of DFPN are higher than the other methods. Furthermore, the F1, mAP, and FPS of DFPN are 26.7%, 20.1%, and 17 fps higher than those of FPN, respectively. Finally, the F1, mAP, and FPS of DFPN are 11.7%, 10.6%, and 26 fps higher than those of FPN6, respectively.

#### 3.3.1. Contributions of the Feature Decoupling Module

As shown in Table 1, FPN1, FPN2, and FPN5 perform better than FPN. In particular, the detection speed of FPN1 runs faster than that of FPN by 16 fps, and the mAP of FPN1 is higher than that of FPN by 10.7%. Because FPN1 increases the weight of the classification score, it increases the proportion of the classification task in the NMS score and improves the network recognition accuracy.

**Figure 8:**
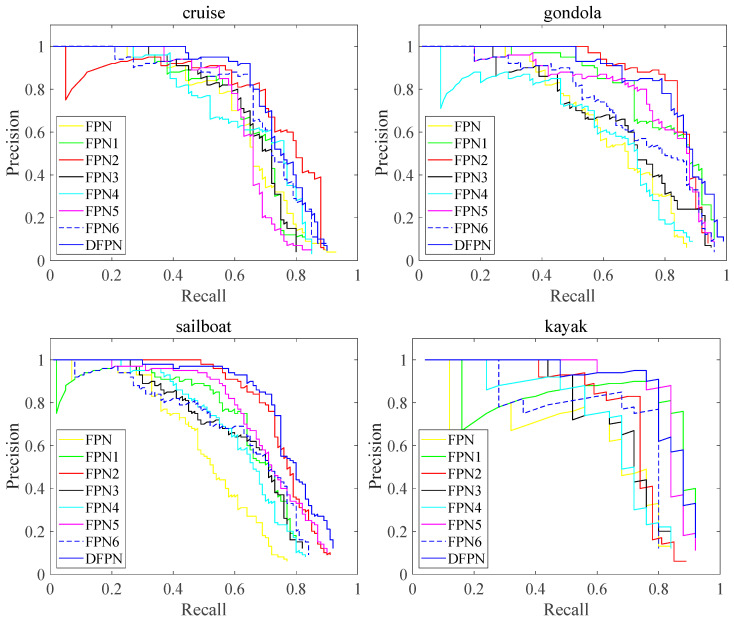
P-R curves of different methods tested on four types of ships.

Figure 8 shows the PR curves of FPN, FPNx, and DFPN tested on four ship types. In order to improve the ship identification recall rate, the NMS threshold needs to be lowered, but this will lead to a decrease in the accuracy rate. When similar features from different ships are mixed together, the classification and localization scores are disturbed, resulting in similar NMS scores from different ships. The above factors lead to the weak ability of FPN to discriminate ships, and its P-R curve drops sharply with the increase in recall. When the recall rate is set to 0.60, the average precision of the four ships of FPN1 is about 83%, and the average precision of the four ships of FPN is about 72%. Moreover, FPN2 and FPN5 have higher map values than FPNx. This shows that the feature decoupling module can solve the problem of consistency between the classification scores and target box regression in ship detection and improve recognition accuracy for similar ships.

#### 3.3.2. Contributions of FPA Module

**Figure 9:**
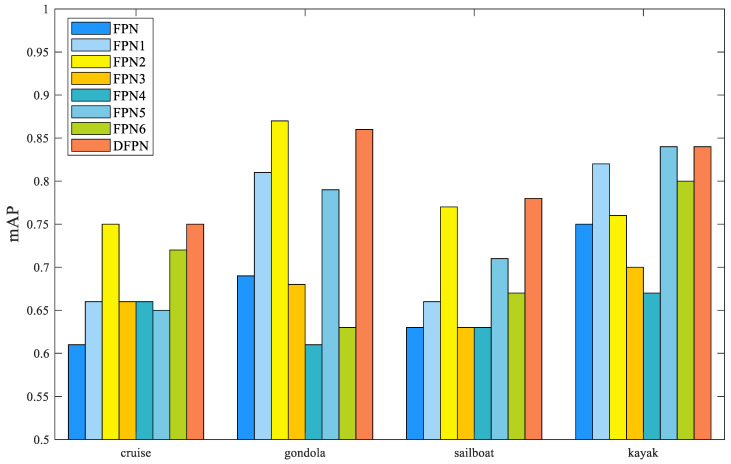
mAP values of FPN, FPNX, and DFPN.

Overall, FPN2 outperforms FPN1 with a 3.8% increase in the F1 score and a 3.6% increase in the mAP score. In Figure 8, the purple curve of FPN2 is compared with the cyan curve of FPN1 in the P-R curve graph. The FPN2 curve is slightly higher than the FPN1 curve in recognizing cruise ships, gondolas, and sailboats. Figure 9a showes that the FPN2 network increases the AP values for cruise ships, gondolas, and sailboats by 8%, 4%, and 10%, respectively. The FPA module fuses attention-enhancing contour features with deep semantic features to improve the accuracy of the network for multi-scale ship recognition. It proves that the FPA module has better adaptability to the different types of ships and improves the accuracy of small ship detection.

As shown in Figure 9, DFPN has the highest AP values for cruise ships, sailboats, and kayaks with values of 0.75, 0.78, and 0.84, respectively. FPN2 has the highest AP value for gondolas with a value of 0.87. Furthermore, it can be observed from Figure 9a that FPN2 achieves higher AP values for cruise ships, gondolas, and sailboats with values of 0.75, 0.87, and 0.77, respectively. In particular, the CBAM valve compensates for the overfitting problem caused by the small dataset when detecting kayaks.

#### 3.3.3. Contributions of Multi-Feature Fusion Method

**Figure 10:**
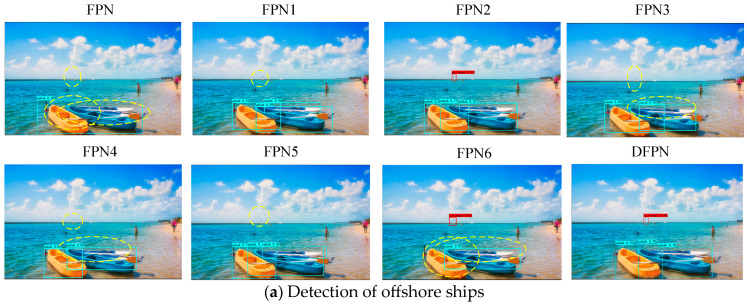

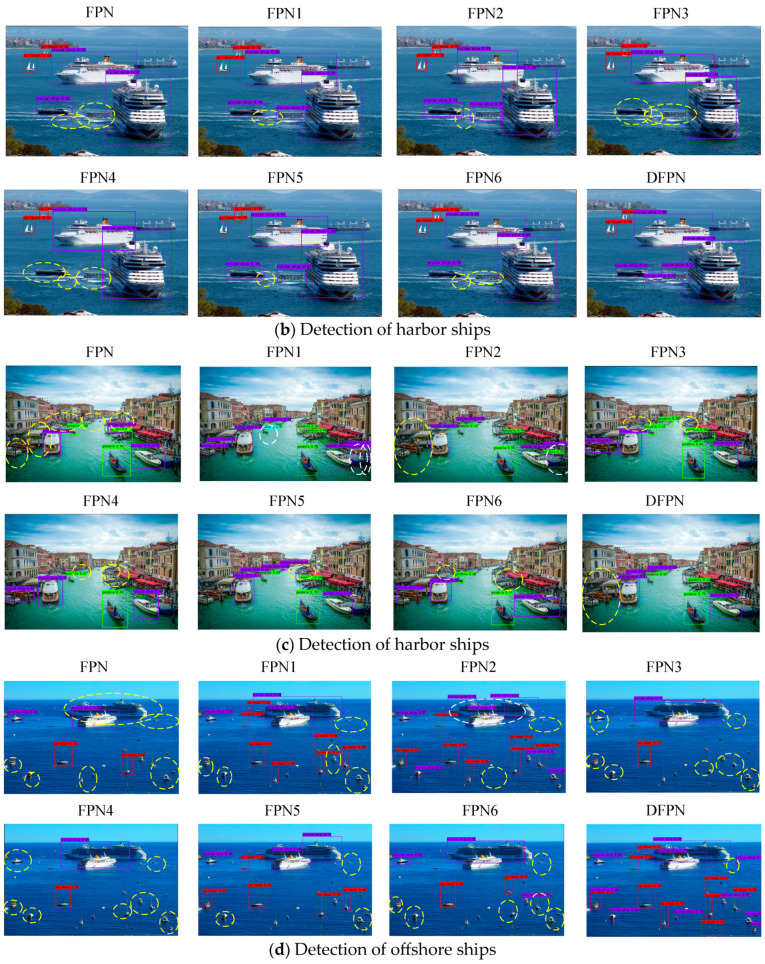
Detection results of FPN, FPNX, and DFPN. The purple box, red box, green box, and cyan box represent the detection of cruise ships, sailboats, gondolas, and kayaks, respectively. The yellow dashed line represents missed detection results.

Table 1 shows that the F1 and mAP of DFPN are 5.2% and 3.5% higher than those of FPN2, respectively. It might be because DFPN is easy to learn discriminative features of multi-scale ships, and redundant features are effectively suppressed by FPA along channel and spatial dimensions. The visual detection results of FPN, FPN1, FPN2, and DFPN are presented in Figure 10. We selected four groups of detection results on harbor and offshore ships. According to Figure 10, some conclusions can be summarized as follows.

First, the feature decoupling module enhances network discrimination for similar features of multi-scale ships. In Figure 10a, FPN1 has much fewer false alarms and higher precision compared with FPN. Second, the weighted loss function improves the training efficiency and adaptability of the network to multi-scale ships. In b, the four types of ships are detected with higher probabilities by FPN1 than those predicted by FPN1. Third, the FPA module concentrates more on ships’ texture and semantic features and obtains representative features of multi-scale ships. In Figure 10c, densely arranged ships in harbor are detected by DFPN, but are missed by FPN2. In Figure 10d, the probabilities of false alarms predicted by DFPN are lower than for the other three methods. DFPN achieves higher ship detection accuracy and recall than FPN, FPN1, and FPN2. The results show that the proposed DFPN method can provide better detection for multi-scale ships by a reasonable combination of the proposed modules and solves the problem of difficult feature extraction for small ships.

### 3.4. Comparative Experiments

In this section, DFPN is compared with four CNN-based methods—YOLOv4, Faster-R-CNN, Mask-R-CNN, and DetectoRS—on the multi-target ship dataset. The parameters used for this comparison experiment are identical to those utilized in the ablation experiment. YOLOv4 and Faster-R-CNN use DarkNet-53 and ResNet-50 as their feature extraction networks, respectively. The same epoch, NMS, learning rate, and weight decay rate are same as those of ablation experiment.

Table 2 shows that the FPS of DFPN is 20, 36, 36, and 35 higher than those of YOLOv4, Faster-R-CNN, Mask-R-CNN, and DetectoRS, respectively. DFPN outperforms YOLOv4 with a 7.2% increase in F1 score and a 6.3% increase in mAP score. This might be because of the well-designed feature fusion and gate attention strategies, which highlight the multidimensional features of ships and adaptively adjust the channel weights of the feature map. Moreover, the weighted loss function in DFPN increases the effective training of the network for positive and difficult samples through the weight parameter, thus improving the accuracy and speed of ship detection. Although Faster R-CNN has a high recall rate, too many error checks affect its ship recognition accuracy. The F1 and mAP of DFPN are 16.3% and 7.6% higher than those of Faster-R-CNN. The reason is that DFPN suppresses the background features by using spatial and channel attention in the FPA architecture.

**Figure 11:**
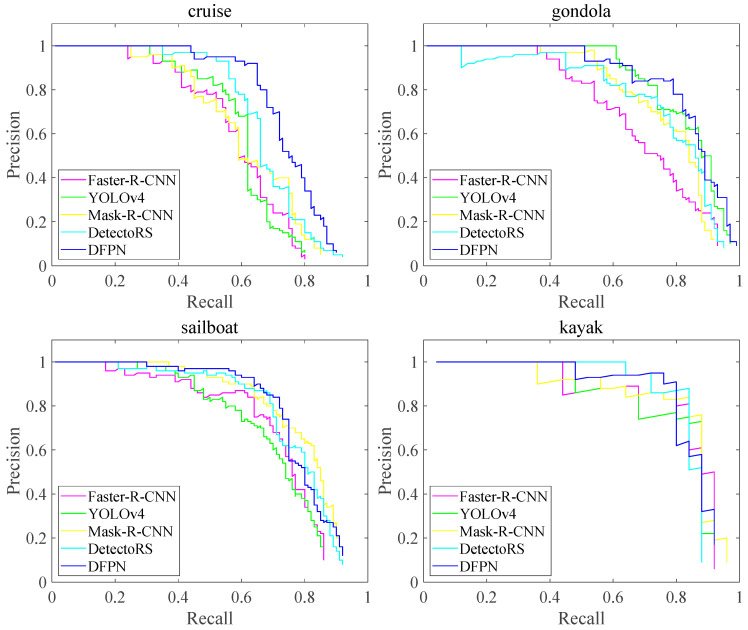
P-R curves of different methods tested on four types of ships.

As shown in Figure 11, it is obvious that the PR curve of DFPN is higher than those of other methods with an increase in the recall rate. When the recall rate is set to 0.6, the average precision of the four ships of DFPN are 11% and 16% higher than those of YOLOv4 and Faster-R-CNN, and 2.2% and 2.5% higher than Mask-R-CNN and Detec-toRS, respectively. In particular, Faster-R-CNN decreases sharply in identifying small target such as gondolas. The reason may be that the feature fusion and FPA module can suppress background information and focus on the ship’s salient features, thus improving the recall and accuracy for ships of different scales.

**Figure 12:**
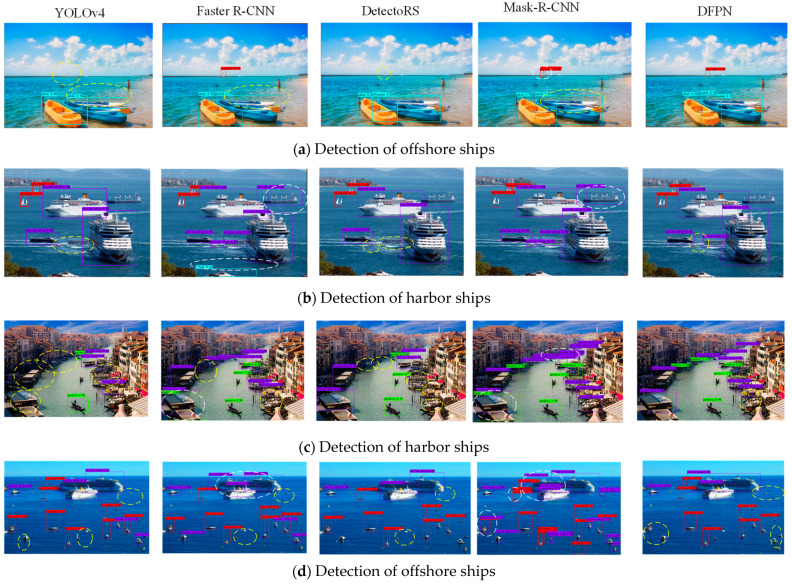
Detection results of YOLOv4, Faster-R-CNN, DetectoRS, Mask-R-CNN, and DFPN. The purple box, red box, green box, and cyan box represent the detection of cruise ships, sailboats, gondolas, and kayaks, respectively. The yellow dashed line and white dashed line represent the missed detection and false detection results, respectively.

The visual detection results of YOLOv4, Faster-R-CNN, DetectoRS, Mask-R-CNN, and DFPN are presented in Figure 12. As shown in Figure 12a, YOLOv4 shows two yellow elliptical dashed boxes due to missing a sailboat and a kayak, Faster-R-CNN shows a yellow dashed box due to missing a kayak, DetectoRS shows a yellow elliptical dashed boxes due to missing a sailboat, and Mask-R-CNN shows a white dashed box leading to a false positive. However, DFPN does not miss any ships and shows better detection capability. In Figure 12b, Faster-R-CNN shows two white dotted elliptical boxes due to two false detections, Mask-R-CNN shows one white dotted elliptical box due to one false detection, and DFPN does not have any white dotted boxes. This is because DFPN concatenates multi-level features to acquire fine-grained semantic information by feature decoupling and the FPA module, which improves the adaptability to multi-scale ships with similar features. In Figure 12c, YOLOv4 has four yellow dashed boxes, Faster-R-CNN has one yellow and one white dashed box, DetectoRS has four yellow dashed boxes, and Mask-R-CNN has two white dashed boxes. The probabilities of missed detections and false alarms predicted by DFPN are lower than the other two methods. In Figure 12d, although the small target vessels are numerous and dense, DFPN can detect them and only a few are missed. Overall, our method not only acquires high precision and recall rates, but also achieves a balance between them. It also demonstrates the effectiveness of our method for detecting harbor and offshore ships.

## 4. Conclusions

In this paper, a decoupled feature pyramid algorithm was proposed for detecting multi-target ships. Firstly, we employed a feature decoupling module based on additive NMS scores to refine ship features into contour features and location features to distinguish ships with similar features. Secondly, a gated attention mechanism was introduced to make the network focus on significant features for detecting ships by reweighting feature maps using channel and spatial attention modules in sequence. Thirdly, a multi-feature fusion algorithm was adopted to generate fine-grained ship features utilizing feature pyramid fusion and contextual information. Finally, a weighted loss function was designed to improve network adaptability for multi-target ship detection. In ablation experiments, we proved the superiorities of our method by exploiting the contributions of every module separately. Compared with YOLOv4, Faster-R-CNN, Mask-R-CNN, and DetectoRS, the experimental results demonstrated that the proposed method has advantages in the detection accuracy and recall rate of multi-target ships.

## Figures and Tables

**Table 1:** Effectiveness of ablation experiments in multi-target ship dataset.

Component	Feature Decoupling	FPA	Multi-Feature Fusion	F1 (%)	mAP (%)	FPS
FPN				50.8	60.8	24
FPN1	✓			65.8	71.5	40
FPN2	✓	✓		72.3	77.3	41
FPN3		✓		62.3	66.8	20
FPN4			✓	61.4	64.4	19
FPN5	✓		✓	68.8	74.8	40
FPN6		✓	✓	65.8	70.3	15
DFPN	✓	✓	✓	77.5	80.9	41

**Table 2:** Comparison table of results of all methods.

Method	Precision (%)	Recall (%)	F1 (%)	mAP (%)	FPS
YOLOv4 [36]	87.2	58.9	70.3	74.6	21
Faster-R-CNN [37]	50.3	73.7	61.2	73.3	5
Mask-R-CNN [38]	52.5	81.3	65.4	75.9	5
DetectoRS [39]	88.9	61.1	71.2	76.8	6
DFPN	93.1	67.2	77.5	80.9	41

## Data Availability

Not applicable.
